# Feeling and performing ‘the crisis’: on the affective phenomenology and politics of the corona crisis

**DOI:** 10.1007/s11097-022-09877-9

**Published:** 2023-01-18

**Authors:** Ruth Rebecca Tietjen

**Affiliations:** Department of Philosophy, Tilburg University, Warandelaan 2, 5037 AB Tilburg, The Netherlands

**Keywords:** COVID-19 pandemic, Crisis, Emotions, Moods, Affective politics

## Abstract

How does it feel to be in a crisis? Is the idea of the crisis itself bound to our affectivity in the sense that without the occurrence of specific emotions or a change in our affective lives at large we cannot even talk about a crisis properly speaking? In this paper, I explore these questions by analyzing the exemplary case of the corona crisis. In order to do so, I first explore the affective phenomenology of crises in general and the corona crisis in particular, thereby paying attention to both individual (personal) and collective (socio-political) crises and crisis experiences. Then, I turn to the limits of the analogy between individual and collective crises. I reflect on how socio-political crises are not simply there but performed and procedurally constructed and show how, in the context of the corona pandemic, fears and hopes, feelings of solidarity and antagonistic emotions mirror political interests and values. While the phenomenological reflections in the first part help us to account for the fact that crises are not just objective facts but also subjective forms of experience, the political reflections in the second part help us to do justice to the inherently political nature of the language and experiences of (collective) crises. I conclude by pointing out a twofold relationship between (socio-political) crisis and critique. Thanks to their characteristic affective phenomenology, crises are tools of criticism; but due to their inherently political character, they also themselves have to be subjected to critique.

## Introduction

The corona crisis has affected us in manifold ways. We may have experienced the virus as a threat to our health and life, especially when particularly vulnerable, and felt helpless or upset when seeing people exhausted, suffering, dying. Those who lost their jobs and income but also those working in draining and underpaid jobs in the health care system may have felt left alone, angry, desperate. As privileged academics, we have been forced to abandon our trips abroad and to cancel or postpone our travel plans to an uncertain future. Enforced collective travel bans have brought rest into our hectic lives. But they also have deprived us of the possibility to meet our colleagues, friends, and family members in person and disrupted our habitual ways of social interaction. For those of us living alone, the enforced social distancing may have brought solitude or loneliness. For those living with others, especially with young children, it may have been a time of cozy intimacy but also of an overburdening load of childcare and work obligations. While the successful collective endeavor of finding vaccines and making them available to large proportions of our society may have given us hope, the rising polarization about corona policies, the globally unequal distribution of access to vaccinations and health care, and the emergence of ever-new variants and waves of the disease may have drawn us into resignation, depression, and despair, or evoked incomprehension, anger, and outrage.

While each of these affective reactions to the corona crisis – alongside numerous others – might be worth philosophical investigation, they all take for granted one thing, namely that the situation we are in is a situation of crisis. This allows us to take a step back and take a synoptic view on the aforementioned affective phenomena, asking what it is that makes them an affective reaction *to a crisis*. How does it feel to be *in a crisis*? Is the idea of the crisis itself bound to our affectivity in the sense that without the occurrence of specific emotions or a change in our affective lives at large, we cannot even talk about a crisis properly speaking?

In this paper, I explore these questions by discussing and analyzing the exemplary case of the corona crisis. In order to do so, I first offer an analysis of the idea and phenomenology of crisis (section 2.1) and then zoom in on the affective dimension of crisis experiences in general and the corona crisis in particular (section 2.2). In my analysis, I especially draw on insights from the phenomenological tradition whose engagement with crises so far has been largely focused on individual, “existential” crises, such as crises in the context of illness or confrontation with death. But, as I show, such insights can also be applied to collective crises like the corona crisis. In the second part of the paper, I reflect on the limitations of the analogy between individual and collective crisis experiences. Drawing on insights from political philosophy and theory, I first offer an analysis of the language of crisis, reflecting on the question of how socio-political crises are not simply a given but performed and procedurally constructed (section 3.1). I then outline the implications of these observations for our understanding of the affective phenomenology of the corona crisis, thereby especially focusing on the two complementary pairs of (collective) fear and hope, feelings of solidarity and antagonistic political emotions (section 3.2).

The structure of the paper mirrors my conviction that in order to gain a full understanding of the corona crisis as a collective affective phenomenon, we need to draw on both phenomenological literature on crisis experiences and crisis literature in political philosophy and theory. While the phenomenological literature helps us to account for the fact that crises are not just objective facts but always also subjective forms of experience, the political literature helps us to do justice to the inherently political nature of the language and experiences of (collective) crises. As the rapid growth and influence of critical phenomenology demonstrates, phenomenological and political perspectives are not only mutually compatible; phenomenology, if conducted in a critical spirit, also offers us unique tools to uncover the quasi-transcendental yet contingent oppressive socio-political structures underlying our experiences (see, e.g., Weiss et al., 2020). The perspective I take in this paper is inspired by critical phenomenology in that I take the plurality of crisis experiences seriously and critically reflect on the question of how even our individual experiences of the corona crisis reproduce, are based on, and entangled with liberating or oppressive socio-political structures.

Before commencing with my analysis, a brief note on epistemic humility and engaged philosophy. In the introductory paragraph and other passages, I am consciously talking about “us” because this article is not written from a detached and impersonal perspective but from the perspective of someone who, like all of us – all of you, I suspect – has been affected by the corona pandemic. It is interested in the manifold ways in which the corona crisis has manifested itself in our individual and collective affective lives. I acknowledge the limitedness of my own idiosyncratic experiences and perspective and the way they mirror my socio-political situatedness and privilege. Nowhere do I intend to assume that our experiences are all the same, nor that I am in the epistemic position to speak for all others. On the contrary, one of the aims of my article is to raise awareness of the plurality of crisis experiences connected to the corona pandemic and the problematic tendency to talk about “*the* corona crisis” as if our experiences were all the same. That being said, I still believe that talking about us and to you is legitimate and appropriate when one wants to practice philosophy in an engaged rather than detached manner, as is my aim here.

## The affective phenomenology of crisis

### The idea and phenomenology of crisis

In order to answer the question of how it feels to be in a crisis, we first need a preunderstanding of what a crisis is. The aim of this subsection is to outline such an understanding. A crisis is a decisive moment or period of time; a turning point or transition phase in which a decision is made or demanded. A reflection on the conceptual history of the term allows us to identify two key features of the modern concept of crisis that, as such, also determine our discourse about the corona crisis. Etymologically, the term “crisis” goes back to the Greek verb κρίνω, meaning to “choose,” “decide,” or “judge.” It originates from the medical realm where it denoted the critical moment of a disease in which it was decided whether the patient would recover or die (Koselleck, 2006). In other words, “crisis” denoted an *objective* condition; a condition whose outcome did not depend on a decision of the patient, doctor, or any other person or collective; rather, it was the disease itself that “decided” the patient’s fate. While examples of this usage still exist today, e.g., in the medical context, we now often conceive of crises as situations that demand decisions and actions – *our* decisions and actions. Moreover, we conceive of them as not purely objective but at least in part subjective conditions – as a form of experience. It is in this context where the question emerges as to how it feels to be in a crisis. Phenomenology, as a branch of philosophy that studies subjective experiences, is uniquely suited to explore this subjective dimension inherent to the modern concept of crisis.

A second shift compared to the original usage is that the concept “crisis” has extended its meaning to all realms of life including the existential, social, political, historical, and economic realms. It is no longer only individual persons who can be in a state of crisis but also social entities, such as the health care system, state, economy, environment, or the sciences and philosophy themselves (Husserl, 2012). In the case of the corona crisis, the most obvious threats we have been facing are threats to our health and lives, the health and lives of our loved ones and fellow citizens, our health care system and the medical sector at large. In this regard, each of us may have experienced the pandemic individually, but the corona crisis is also an inherently social and collective phenomenon. This explains why, in order to understand the (affective) phenomenology of the corona crisis, we need to consider both its individual (existential) and collective (socio-political) dimension, and how both dimensions interact.

The idea that crises *demand* actions (Milstein, 2015) presumes that there is something that we ourselves can do about the outcome of the situation. It depends on us, individually or collectively, whether we will live or die, flourish or perish. In the case of the corona crisis, governments saw themselves confronted with the need to take action in order to prevent their health care systems from collapsing and protect our social structures at large. Moreover, especially in the socio-political realm, the idea of crisis often involves the presumption that the emergence of the crisis itself is (at least in part) the effect of human behavior, action, or omission. Crises signify failure. Although the occurrence of a novel virus such as SARS-CoV-2 and a highly contagious new disease that in a significant number of cases is severe or even deadly, such as COVID-19, resembles a natural catastrophe, there still have been extensive debates about the question to what extent political decisions and socio-political structures, such as (to give some random examples) the way we live alongside, sell, and consume animals in our globalized world, the Chinese information policy in late 2019 when the first patients died, or nationalistic forms of protectionism when the pandemic hit Europe, contributed to the development of this event into a crisis of global proportions (see, e.g., OECD, 2022 for an evaluation of policy responses to the COVID-19 crisis in OECD countries). In other words, both the existence of and solution to the crisis have been experienced as dependent on human behavior and action. This is the context in which the concept of crisis obtains an ethical meaning, invoking “a moral demand for a difference between the past and the future” (Roitman, 2012). More generally, the focus on responsibilities for the emergence, development, and resolution of crises defines “crisis” as a “formula legitimating action” (Koselleck, 2006, 368).

In crises, not just anything is at stake; it is something of utmost importance to us, our individual or collective well-being, or even our existence (McConnell, 2020). In the medical context, the decision is a life and death one for the patient. Similarly, the corona pandemic has threatened core values – our health, lives, and health care system – of our individual and collective existence. But it need not be our individual or collective existence, literally speaking, that is endangered; it may also be the meaning, value, significance, or truth of our lives that is at stake. Here we can discern traces of the religious meaning of the term, the idea of the crisis as a divine judgment and, in particular, the Last Judgment, i.e., the final decision between salvation and damnation (Koselleck, 2006, 371). In a more mundane reading, crises are situations in which our ordinary way of being-in-the-world and being-together are put into question. They demand reorientation, decision, and action. Due to what is at stake in crises, they come with an air of urgency and may require struggle, sacrifice, and extraordinary – or even “extreme” – action (Roitman, 2012; McConnell, 2020; Berger, 2018, 75–112). This explains why the notion of “crisis,” as I will elaborate in the next section, is a key element of populist and extremist – but also revolutionary – rhetoric.

Finally, crises typically have a violent character. By “violent” I mean that they are disruptive and experienced as a form of suffering forced upon us. However, the experience of suddenness, force, and suffering is not without ambiguity. First, the process leading up to a crisis can also be a slow one, for example, the enduring failure of communication preceding a marital crisis or the century-long destruction of our environment that has led to the climate crisis (McConnell, 2020). So, the suddenness of crises in the first place is a “suddenness of appearance” (an epistemic suddenness) (Wang, 2014, 261), rather than an ontological suddenness (a “suddenness of existence”). In the context of the corona crisis, for instance, there were early warnings in December 2019 and January 2020 (see, e.g., WHO, 2020); however, individually and collectively, it took many people a few more weeks to realize that we were dealing with a crisis of global proportions affecting them, personally, rather than just others. Moreover, as noted above, although crises are typically experienced as something forced upon us, it can still be we ourselves who have caused the crisis, contributed to its emergence and development, or failed to be prepared for its arrival. Finally, as I explain in more detail below, although crises necessarily involve an element of suffering and threat, the experiences of crises may also involve neutral, ambiguous, or even positive elements. For example, while the corona crisis may have severely restricted our movement and potential in certain ways, it also prompted us to revisit or take up new leisure activities, explore and appreciate our local environments, and made us aware of what really matters to us (see, e.g., Carel & Kidd, 2020).

To summarize, crises are “big” – i.e., they concern what really matters to us – “bad” – i.e., they involve suffering – and “urgent” – i.e., they require immediate action (Boin et al., 2009, 86). Importantly, phenomenological and conceptual analyses here are directly connected because the experiential dimension is itself an inherent dimension of our concept of crisis. Crises violently disrupt our ordinary way of being-in-the-world and being-together through putting into question what we ordinarily take for granted and confronting us with a threat to core values of our individual or collective existence.

To distinguish different types and tokens of crisis, it is important to note that crises have subjects; something that is “in crisis.” Different types of crises can be distinguished in virtue of the questions of what it is that is in crisis, what it is that is put into question, and what kind of decision and actions are demanded. Personal (existential) crises demand personal decisions and actions, collective (social, political, economic) crises demand collective decisions and actions. For example, in an existential crisis (e.g., caused by a life-threatening illness), our individual form of life and self-conception are put into question and call for reconceptualization and reformation. In a social crisis (such as the so-called “European migrant crisis”), it is our social form of life and collective self-conception, in a political crisis (such as the so-called “crisis of representation”), our political system, in an economic crisis, our economic system that are put into question and demand reconceptualization, reconstitution, reformation, or even, in the most extreme case, revolution. As a crisis of health and the health care system, the corona crisis has affected both our individual ways of being-in-the-world and our being-together as a society. It has been experienced on both the individual and collective level. By contrast, other crises, such as severe illnesses or midlife crises, are mainly individual although they also affect and concern the person’s social life and the socio-political structures in which they are embedded. Still other crises are both collective and individual but only severely affect a small proportion of the population.

But what exactly is it that we are talking about when talking about the “corona crisis”? On the personal level, the corona crisis has put into question what we ordinarily take for granted, for instance, that we can plan our future, spend time with our colleagues, friends, and family members, get adequate medical treatment when in need of it, won’t catch a life-threatening disease when leaving home, won’t die – at least not tomorrow, or this year, or this decade (see, e.g., Aho, 2022a; Carel & Kidd, 2020). But those of us who suffered from severe forms of the disease may also have experienced a crisis in the original, medical sense of the term. For them, the crisis was a matter of life or death. Still others are suffering from long COVID; have seen their loved ones suffering or dying; have seen themselves stigmatized and excluded; have suffered psychologically as a result of the pandemic, through experiences of grief, loneliness, or anxiety; have lost their jobs, or been exposed to domestic violence.

On the collective level, in the first place, it was our health care systems that were severely strained and at risk of collapsing. Governments declared “states of emergency,” allowing them to take extraordinary measures to confront the crisis (see, e.g., Crego & Kotanidis, 2020). But not only our health care system, but also large proportions of our social lives in the domains of education, work, and culture have been profoundly reshaped by the pandemic through school closures, remote working, and closed cultural institutions. This and more specific politically controversial pandemic measures, such as lockdowns, curfews, mandatory masking or vaccinations, have led to massive and sometimes violent political protests, crystallizing, among other things, on the question of whether the corona pandemic indeed (still) should be considered and treated as “a crisis.” Instead, in a rhetorical inversion, some – e.g., supporters of the German protest movement “Querdenken” – have diagnosed a “crisis of representation,” where it is no longer the pandemic but the (alleged) exploitation of the crisis by our media and governments that constitutes the *real* crisis. Going even further, protest movements like these have themselves been interpreted as symptoms of a political crisis.

In other words, there is not just one, but a plurality of COVID-19 experiences and maybe even of crises themselves. Even if we might still say that these diverse experiences *together* constitute the singular subject of the corona crisis, talking about the COVID-19 pandemic as a crisis is highly ambiguous in that it leaves open which of these experiences are included and which are excluded. Denying the plurality of crisis experiences in the context of the corona pandemic by appealing to “We are all in the same boat” metaphors can be highly problematic and even offensive, especially when put forward by the privileged. Not only do they risk denying existing injustices by ignoring the difference that their position of privilege makes in how they experience the pandemic; they are also at risk of reinforcing injustices by placing a disproportionate burden on those who are already worse off to ameliorate the situation.

### The affects of crisis

In the previous subsection, I introduced the concept of crisis and argued that crises violently disrupt our ordinary existence through putting into question what we ordinarily take for granted and confronting us with a threat to core values of our individual or collective existence. What does this analysis imply for the question of affectivity? How does it feel to be in a crisis? The aim of this section is to discuss these questions, thereby particularly focusing on the case of the corona crisis and reflecting on the question of how specific affective changes, emotions, and moods are intimately connected to the concept of crisis itself, as I have developed it in the previous section.

Here and in the following, I use the concept of affectivity as an umbrella term covering all sorts of affective phenomena including those of emotions, moods, and atmospheres (for an introduction, see, e.g., Deonna & Teroni, 2012). Following a major line in contemporary philosophy of emotion, I take emotions to be representational affective states of mind that evaluate an object or situation in the light of what we care about (see, e.g., Helm, 2001; Roberts, 2003). Moods (or, as they are sometimes called, existential feelings), by contrast, are pre-intentional feelings that constitute, restrict, and infuse spaces of possibilities (Ratcliffe, 2008). Whereas emotions are directed toward (more or less) specific objects in the world, moods are directed at the world at large or our existence as a whole. They are ways of being-in-the-world that, among other things, determine what kind of emotional relations to the world are available to us.

First, given that crises violently disrupt our ordinary way of being-in-the-world and being-together, what is characteristic of crises, in the first place, are not specific emotions but a change of our affective lives at large, including a change in the type, intensity, depth, content, form, and/or constellation of our emotions. The change reflects the temporal character of crises, the fact that in crises what we ordinarily take for granted is no longer the case. This provokes emotional reactions, such as, for example, fear of losing one’s job due to the pandemic or a renewed sense of solidarity with one’s neighbors. But it also provokes shifts on a more profound level, namely the level of our moods, that reflect our altered sense of (im)possibility. The corona pandemic has put into question what we ordinarily take for granted, for example the functioning of our health care system or the existence of a public space in which we can freely come together as embodied beings. While the breakdown of these structures has always been theoretically conceivable, the pandemic has made it an affectively salient, “real” possibility. Importantly, the affective reactions to the pandemic thereby mirror our socio-political situatedness. For instance, taking for granted the accessibility of health care or the public space is itself a privilege that, as such, is not accessible in the same form, for example, to illegal immigrants.

Second, nonetheless, there are typical affective experiences of crisis that reflect the characteristic elements of what it means to be in a crisis, among them fear and anxiety as a reaction to impending dangers, helplessness in the face of uncertainty, and experiences of shock, disorientation, vulnerability, and loss mirroring the disruptive violation of the ordinary. Indeed, in the case of the corona pandemic, all these feelings have been observed (see, e.g., Froese et al., 2021). Especially, the pandemic has been described as a situation that has evoked a Heideggerian sense of uncanniness (Aho, 2020), anxiety (Trigg, 2022), collective disorientation (Ratcliffe, 2021; Velasco et al., 2021), loss of trust (Lopes, 2021), fear (Degerman et al., 2020), grief as a reaction to bereavement and other forms of loss (Richardson et al., 2021), and loneliness (Aho, 2022b). Whereas some of these phenomena are emotions, others are more profound and often more encompassing and enduring; they are moods rather than emotions.

Since the concept of crisis is dynamic, these affective experiences themselves typically change over time. Whereas the first reaction to a crisis may be fear, helplessness, and disorientation, over time we reorient ourselves, and, depending on how we are coping with the crisis, our affective states will change. For example, in the case of the corona pandemic, whereas first, experiences of fear and anxiety may have dominated our affective lives, later, frustration, resignation, and anger became more prominent. This in part can be explained by the fact that no longer only the pandemic itself but also pandemic management or – depending on the perspective – mismanagement as well as the behavior and reactions of our fellow citizens to the pandemic have become an object of affective engagement.

Moreover, there are characteristic affective experiences connected to specific types of crises, for example, nostalgia for lost opportunities as a characteristic element of a midlife-crisis (Setiya, 2017), anger and outrage against “those in power,” “the elite,” or “establishment” held responsible for “the people’s” grievances as prototypical populist emotions (Moffitt, 2015; Rico et al., 2017; Tietjen, 2022), or loneliness as a reaction to trauma (Brison, 2003; Stauffer, 2015). In cases like these, the affective states in question are indicative of both the fact that we are in a crisis and the kind of crisis we are in. The variety of affective reactions connected to the corona pandemic – reaching from fear, anxiety, and uncertainty, to nostalgia, grief, and loneliness, to anger and outrage – as well as the way our affective reactions are embodied and socially embedded (see, e.g., Aho, 2022b; Degerman et al., 2020; Trigg, 2022) – especially in the case of a pandemic that both threatens our bodies and represents bodies as a threat – supports the conjecture that what we are dealing with in the case of the corona pandemic in fact is a plurality of crises.

Third, the affective experiences of crisis often come with a characteristic epistemic “depth” (Cataldi, 1993; Mendonça, 2019; Pugmire, 2007). In existential crises, we are confronted with basic constituents of human existence that in our ordinary being-in-the-world remain hidden. This may, for example, be our vulnerability, morbidity, and mortality, as is the case in a serious illness (Carel, 2016; Tietjen, 2021), it may be unfulfilled religious desires and needs, as is the case in a spiritual crisis (James, 2002; Roberts, 2007, 37; Tietjen, 2014), or specific features of our lives as temporal beings, as is the case in a midlife-crisis (Setiya, 2017). In socio-political crises, we are confronted with basic structures of our social lives that we ordinarily take for granted but that – as the crisis reveals – are not so stable. On the existential level, our affective reactions to the corona crisis, for instance, may have pointed us to our vulnerability and mortality, but also to our social nature and the fact that we need others in order to be and become ourselves. On the socio-political level, it has confronted us with the fragility of our health care systems and European solidarity. More generally, the idea of the crisis is connected to the idea of a “moment of truth” in which the true nature of ourselves, our society, our economy, our health care system, or whatever it is that is in crisis is revealed (Roitman, 2014, 4).

Fourth and relatedly, the affective experiences of crisis come with a characteristic practical depth. Both our *understanding* of ourselves and the world and our *being* ourselves, *being*-together, *being*-in-the-world are transformed. On the individual level, this change can be characterized as a change of our moods. On the social level, it can be described as a transformation of our collective moods or atmospheres (Osler & Szanto, 2022; Ringmar, 2018; Trcka, 2017). As *collective* affective states of mind, they are characterized by the fact that the affective experiences of the collective’s members resonate with each other. As collective *moods*, they constitute, restrict, and infuse shared spaces of possibilities. As an individual and collective crisis, the corona crisis has reshaped both our individual and our collective moods, and together with them our more specifically directed emotions, cognitions, and actions. This is attested to by the analyses cited above that describe our reactions to the corona pandemic in terms of existential feelings of uncanniness, anxiety, disorientation, loss of trust, and loneliness, and, especially, in terms of *collective* feelings, moods, or atmospheres rather than in terms of (individual) emotions (Aho, 2020, 2022b; Lopes, 2021; Ratcliffe, 2021; Trigg, 2022; Velasco et al., 2021).

As epistemically and practically deep feelings, the affective experiences of crisis are of potential epistemic and practical value. On the epistemic level, they allow us to become aware of basic features of human existence, of our social or political life (Carel, 2016, 204–228). In doing so, they open up the possibility of rethinking and reshaping our individual life and the structures of our society. In confronting us with our facticity, existential crises allow us to relate differently to the basic constituents of our existence, to rethink our values and expectations. Therefore, they have transformative power. For example, it has been argued that “illness can also be an opportunity and a challenge that bring about edification and personal growth” (Carel, 2016, 131; see also Cholbi, 2021; Kidd, 2012; Kidd & Carel, 2020). It does so, among other things, through revealing hidden abilities, inviting more intimacy in our relationships, changing our values and priorities, and making us focus on the present moment. Similar effects have been attributed to the COVID-19 pandemic (Carel & Kidd, 2020). For example, it has been described as an opportunity to rethink our lives, (re)appreciate the importance of family and close friends, and renew our sense of solidarity. Socio-political crises confront us with the contingency of the basic structures of our societies. We no longer take them as a given but recognize them as subject to potential change. This opens up a space for re-evaluation and critique. Therefore, socio-political crises have critical and emancipatory potential. Again, such a potential has also been ascribed to the COVID-19 pandemic (Carel & Kidd, 2020). For example, it has been portrayed as an invitation to rethink our travel practices, to value the contribution of key workers in various sectors, and to critically reflect on the structures of our health care systems. Together, this constitutes crises in general and the corona crisis – or *crises* – in particular, as situations that harbor both threats and opportunities for us as individuals and as a society.

To summarize, the idea of crisis as a decisive moment or period of time in which our ordinary way of being-in-the-world or being-together is put into question, core values of our individual or collective existence are threatened, and a decision is made or demanded is intimately connected to a specific shift in our affective lives. Since what is “ordinary” depends on the person and their socio-political situatedness, so too does the way in which different people have experienced the corona pandemic. Still, there are typical affective reactions to crises that mirror what it means to be in a crisis. In the context of the corona pandemic, they include feelings of fear, anxiety, shock, disorientation, and vulnerability as a reaction to threat and the violent disruption of the ordinary, but also social and moral emotions, such as anger and indignation, as soon as the pandemic and its management became a more politicized issue, that reflect the ethical dimension of the concept of crisis. It is these more specifically directed emotional affective states that I turn to in the next section when reflecting on the affective politics of the corona crisis.

## The politics of crisis

### The political language of crisis

Up to now, I have treated the concept of crisis as a purely descriptive concept, as if crises simply were a given. However, in the socio-political realm, the concept of crisis fulfills both an explanatory and a justificatory function – it justifies actions. Seemingly innocent ones like allocating funding for academic research projects; more drastic ones like restricting freedom of movement; and highly controversial ones like declaring a state of emergency that allows prime ministers to rule by decree and suspend democratic decision-making and control mechanisms. Challenging established ways of political decision-making and action is inherent to the concept of a socio-political crisis itself. As states of emergency that confront us with a threat to the functioning of essential sectors of our society (e.g., our health care system, economy, or democracy) – and, together with it, a threat to our individual and/or collective well-being or even existence – crises do not offer the luxury of dissent and critique, or so the logic goes. Rather than protracted processes of democratic deliberation and decision-making, they demand immediate, determined, collective actions (Tietjen, 2022).

Remarkably, the idea of the crisis belongs to neither a specific position in the political power spectrum nor a specific ideology. It can be used by both oppositional forces, seeking a change of policy or to take power, and the government, striving to consolidate power (for the following, see McConnell, 2020). Governments frame threats as “crises” to align different parties around a common goal, thereby bracketing political disagreement. They use the language of crisis to attack political opponents for their lack of solidarity in a state of exception, to silence dissent, and justify extraordinary action. In juridical terms, these functions are inscribed in the tool of the “state of emergency.” But the language of crisis can also appeal to a sense of opportunity and the vision of a different – *better* – future. Oppositional parties, lobby groups, social movements, and media use the language of crisis to disturb political routines, attract attention, and promote radically different opportunities and policies. They present policy sectors or the government at large as being “in crisis” in order to promote political change. Hence, the framing of crises is itself an important element of the political struggle for power (Boin et al., 2009).

Moreover, the language of crisis can be employed by parties from all positions on the ideological spectrum, from left to right, progressive to conservative, democratic to anti-democratic. For example, in his first inaugural address in January 2009, Barack Obama claimed that “we are in the midst of crisis” (Obama, 2009). Ecological activist Greta Thunberg, in her speech at the World Economic Forum in Davos in 2019, asked us to feel and “to act as you would in a crisis” (Thunberg, 2019). But right- and leftwing populists also regularly appeal to our sense of crisis, thereby interpreting crises of specific policy sectors as symptoms of a more profound “crisis of representation” (Moffitt, 2015). Despite all differences, in all these cases, the appeal to experiences and feelings of crisis fulfills the function of creating a sense of urgency, criticizing the hegemonic social or political order, unifying people across existing social groupings, mobilizing them to action, and promoting the vision of a different, better future. But crisis rhetoric is not confined to democratic agents. It is also a tool of violent extremists (Berger, 2018, 75–112). Construing an out-group as an intrinsic threat to their in-group, they call for the exclusion, domination, or elimination of the out-group. Here we exemplarily see how the language of crisis – if it involves elements of blaming, shaming, and portraying others as essentially evil – can turn into an anti-democratic weapon.

Crisis rhetoric thus comes with at least two democratic risks: silencing dissent and developing into anti-democratic antagonism. Applied to the COVID-19 pandemic, the danger of democratic backsliding that is tied to the political language of crisis invites a careful observation of the crisis rhetoric (and especially *prolonged* crisis rhetoric) employed by governments but also of the inverted crisis rhetoric of oppositional parties (and especially populist and extremist oppositional parties). Put in more positive terms, the commitment to social democracy and, even more, democratization obliges us to use the language of crisis in a way that expands democratic values. Above all, it calls us to avoid any form of othering that leads to a hostile antagonism. But it also obliges us to not just manage the crisis but also work towards a future in which pre-existing injustices that have been revealed by the pandemic, such as health inequality and lack of democratic representation, are mitigated.

### The affective politics of crisis

What does this imply for the affective phenomenology of crises? First of all, even our individual experiences of the corona crisis (or crises) are socio-politically situated. For instance, experiencing the pandemic as uncanny presupposes that before the pandemic we somehow felt “at home” in the world – a presupposition that not all of us share. Even beyond that, our affective reactions are shaped through our interaction with other people. Although this is certainly true for all our affective reactions – not just those related to the corona pandemic – reflecting on the socio-political fabrication and contingency of our affects is particularly relevant in a context in which the appeal to our affects serves the function of justifying such drastic actions as it does in the context of socio-political crises. This reflection helps us to better understand both how our affective reactions to the corona pandemic mirror political convictions and how they serve the purpose of justifying political actions. Indeed, the idea of immediate, determined, collective action – that, as I have argued, is an inherent part of the idea of socio-political crises – is itself closely connected to the domain of emotions and, more precisely, the domain of shared emotions (Laclau, 2007). Collective crisis experiences thereby span the poles of fear (or anxiety) and hope (trust or confidence), feelings of solidarity and antagonistic emotions. Importantly, although these are complementary pairs, they are not mutually exclusive. We can be hopeful and fearful at the same time, for example in “fearful hope,” i.e., when we hope that some feared event will not occur (Stockdale, 2021, 26). Or we can be solidary and yet also antagonistic, for example in “false solidarity,” i.e., when in-group solidarity is generated through outgroup hostility (Szanto & Slaby, 2020, 489).

The first pair of affective phenomena, that of fear and hope, mirrors the fact that crises are decisive moments in time that harbor both threats and opportunities (on the politics of hope, see, e.g., Blöser et al., 2020; Stockdale, 2021; on the politics of fear Robin, 2006; Nussbaum, 2018). Although typically both elements are present, one of them can be or become dominant in the discourse and experience of crisis. Whereas a newly elected president may be well-advised to primarily appeal to the visionary dimension of moments of crisis, appealing to our sense that together we can bring about a better future, a pandemic like the one we have been facing in the past two years seems to be more naturally connected to the fearful apprehension that if we do not act in concert now, something disastrous will happen. At best, it seems to allow for fearful hope. Whereas the emphasis on fear typically calls for a restoration of the status quo ex ante, the focus on hope calls for its substantive alteration (Boin et al., 2009, 84–85). In this regard, fear seems to be a conservative emotion. However, there may also be other reasons to emphasize one or the other emotion. For example, climate activist Greta Thunberg favors fear over hope, not because she wants us to return to a mythological past in which we allegedly still lived in harmony with the natural world but because she believes that fear is the more fitting and motivationally compelling emotion. In other words, it is both epistemic and practical reasons that explain her preference for fear. Similarly, the all-too-optimistic hope for or even belief in racial progress has been criticized for making us blind to past and ongoing racial injustices; instead of hope simpliciter, a “melancholic hope” has been advocated (Winters, 2016).

Much more than with hope, trust, faith, and confidence, the COVID-19 pandemic has been associated with feelings of fear, anxiety, distrust, and even mass hysteria. As described above, anxiety and collective distrust describe our altered way of being-in-the-world. As pre-intentional existential feelings, they do not give rise to questions of epistemic fittingness and appropriateness. “Panic” and “hysteria,” by contrast, are often used as normative terms that presuppose the unfittingness or inappropriateness of the affective reaction in question. Fear lies between these two extremes. As an emotion, it is subject to measures of fittingness and appropriateness, but the ascription of fear leaves open whether it is fitting and appropriate or not. In terms of fittingness, we can ask whether the pandemic indeed instantiates the evaluative property of “being dangerous” – and, in this regard, merits fear – and whether our fear is proportionate to the threat we have been facing (D’Arms & Jacobson, 2000). In terms of prudential or instrumental appropriateness, we can ask whether fear has helped or prevented us from protecting and realizing individual and collective values such as health, freedom, and equality, for instance, by motivating us to comply with drastic measures taken to contain the pandemic and act in concert (Degerman et al., 2020, 2). In moral terms, finally, we can ask whether our fear has been morally appropriate. This includes reflections on the moral content of our emotion but also on our moral character that is revealed by it. For example, we can ask whether the fear of ethnic minorities with a statistically higher risk of becoming sick and severely sick is morally inappropriate due to how it is blind to and reproduces underlying structural injustices.

So, even if, in the first place, fear and maybe even intense fear might seem like a fitting and proportionate reaction to the pandemic given what was at stake, this does not imply that *overall* it was epistemically or morally valuable. For example, even if only considering the values of health, life, and well-being, we may wonder what effect the suspension of other health care services and pandemic measures had on these values. Moreover, we may wonder whether our pandemic fear has become so dominant that it has prevented us from paying attention to and perceiving other, seemingly more remote, dangers to our core political values. So, even if we presuppose that our fear was initially a fitting reaction, its epistemic and instrumental value may remain ambiguous. Things become even more complicated when we add moral considerations into the mix. They reveal that even if fear seems like a natural reaction to a situation like a pandemic, the exact form that our reaction takes is not without alternatives nor is it politically innocent. For example, our collective affective reaction to the pandemic seems to have been largely shaped by the desire to survive and return to how things were before – the desire to restore our seemingly innocent pre-pandemic existence – rather than, say, by the collective hope to reshape our world so as to make it a more just place – a place in which those worst off are better off in a future pandemic, for example. To say this is not to deny the severity of the pandemic. It is to criticize and reject the depoliticization of crisis experiences and politics that come with the air of being without choice. Not only has our fearful reaction to the pandemic been shaped by and mirrored (our and other people’s and institutions’) political interests and values; it also has transformed them (for good and/or bad) (Steinert, 2020).

Like collective crises, individual crises span the poles of fear and hope (see, e.g., Wang, 2014). By contrast, the poles of solidarity and antagonism are tied to the domain of collective crises, although individual crises can have an ambiguous effect on our social relationships, too, fostering intimacy on the one hand and alienation on the other. Feelings of solidarity and antagonistic emotions mirror the inherently social nature of collective crises. They respond to how we, collectively, have been affected by the crisis but also whom we (collectively) take to be responsible for the emergence and development of the crisis as well as its solution. By talking about feelings of solidarity and antagonistic emotions, I do not mean to reduce solidarity or antagonism to affective phenomena. Both solidarity and antagonism for sure are *more* than just affective phenomena and maybe do not even necessarily involve an affective dimension, but here it is the affective dimension of (some or all forms of) solidarity and antagonism that I focus on (on the affective dimension of solidarity, see Müller, 2020; Salmela, 2015; on antagonistic emotions, see Brogaard, 2020, 1–40; Tietjen & Osler, forthcoming).

Whereas a focus on shared grievances and threats to our individual and collective well-being may primarily have a solidarizing effect, a focus on the unjust distribution of suffering and the people, groups, or structures held responsible for this injustice fosters antagonistic emotions, including those of envy, anger, indignation, or even hatred. At first sight, the manifestation of collective affective experiences of crisis either in feelings of solidarity or in antagonistic emotions seems to mirror two ways of building political alliances either through what we have in common or through dissociation (antagonism) (Marchart, 2007, 38–44). But it is important to keep in mind mixed phenomena in which our in-group (and, together with it, our solidarity) is in part defined and constituted by our opposition to others. In the context of the COVID-19 pandemic, we could observe this, for example, in renewed forms of nationalistic protectionism. Moreover, practices of public shaming have powerfully shown how an appeal to “solidarity” can itself be used as a political weapon to foster in-group cohesion and out-group hostility. Finally, as not all forms of solidarity are good, so not all forms of antagonism are bad. At least sometimes, our anger or indignation may have correctly identified injustices and, therefore, have been a fitting or even appropriate reaction to our situation.

Concerning the relationship between the two pairs of affective reactions, it has sometimes been stated that fear is closely tied to in-group favoritism and out-group hostility. For example, there is a correlation between the perception of threat, increased concern with self-protection values, like security and tradition, and a decreased importance of self-transcendence values, like universalism and benevolence (Steinert, 2020). However, again, it is important to note that the question of whether we primarily feel protectionist antagonistic emotions as a reaction to a crisis or whether the crisis has a more global solidarizing effect, is not just a matter of psychological necessity. It is not obvious why the correlation between fear and a decreased importance of self-transcendence values should hold for collective forms of fear that are based on a concern for our collective rather than our individual well-being, nor is it clear that the collective in question necessarily needs to be defined in exclusionary terms, e.g., as one’s nation. The political task for a universalist thus is not necessarily to overcome pandemic fear altogether; they can also try to reshape fear so as to transform it into a collective and inclusionary emotion.

All this demonstrates that our collective feelings of crisis – or our lack thereof – are not politically innocent. They are always already infused with power interests and political values. This points to an important disanalogy between individual and collective crises. Socio-political crises – and, together with them, the experiences and feelings of crisis – are not simply a given. They are socially constructed. To say that crises are socially constructed does not mean to deny their existence. Nor does it mean to deny the objective reality of certain facts underlying crisis experiences, such as the existence of SARS-CoV-2 and its disastrous effects on our health and health care system. Metaphysically speaking, we need to distinguish between certain physical events and facts (e.g., the emergence of a novel virus); our affective reactions to these events and facts; and our categorization of the situation as one of crisis. To say that crises are socially constructed means to say that crises, in a socio-political context, do not exist independently of our awareness and thematization of them (see, e.g., Boin et al., 2009, 83–84). We have to pay attention to how people experience, perceive, and conceive of their own state as well as of what they *do* by calling something a crisis. This is why – as should be clear by now – a philosophical analysis of crises requires the tools of both phenomenology and political philosophy.

## Conclusion

In this paper, I have explored the question of how it feels to be in a crisis through analyzing the exemplary case of the corona crisis. First, reflecting on the etymology of the term “crisis,” I have argued that the concept of crisis itself is (at least in part) a phenomenological concept – i.e., a concept that denotes a specific type of (individual or collective) experience. A crisis is a decisive moment or period of time in which our ordinary way of being-in-the-world or being-together is painfully disrupted and our core values are threatened; it is a situation that demands individual or collective action. In our affective reactions to the corona pandemic, the painful disruption of our ordinary individual and collective existence has manifested itself in a transformation of our affective lives at large. Especially, the threat to core values, such as our lives and health, and violent disruption of the ordinary have manifested themselves in feelings of fear, anxiety, uncanniness, a loss of trust, and feelings of vulnerability. Despite their primarily negative hedonic valence, as epistemically and practically deep feelings, our affective reactions to the corona pandemic still harbor a critical and emancipatory potential on both the individual and collective level. They have made us aware of aspects of our lives that ordinarily remain hidden – e.g., our individual and collective vulnerability – and values and structures that we usually take for granted – e.g., our health and the availability of health care.

While these reflections capture an important part of our affective reactions to the corona pandemic and their importance for our individual and collective lives, they remain incomplete. The idea that crises demand (individual or collective) action directly links them to the domain of action – and in the context of collective crises, such as the corona pandemic, political action especially. Whereas moods may also play a role here, it is collective emotions that direct our actions. This in part explains why channeling our unspecific and not yet clearly articulated affects and transforming them into more specifically directed emotions is of such crucial importance to politics in general and crisis politics in particular. Reflecting on the complementary pairs of fear and hope, solidarity and antagonistic emotions, I have pointed out that our affective reactions to the corona pandemic mirror and then reinforce political stances, and may be based on and support oppressive or liberatory political aims.

There is thus a twofold relationship between crisis and critique. First, crises are a tool of criticism. Experiences of crises are of potential epistemic and practical value. They allow us to become aware of features and structures of our individual or collective existence that in our everyday experience remain unrecognized. Such experiences allow us to relate to our social world anew and, in doing so, harbor the potential for practical transformations. However, as inherently political phenomena (political tools), crises also have to be subjected to critique. Not only do diagnoses of crisis rely on normative standards; to say that something is “in a crisis” means to say that something is amiss with it. Diagnoses of crisis also serve political aims; to say that something is “in a crisis” is to motivate and justify distinctive modes of political decision-making and action, as well as specific decisions and actions themselves. The “opportunities” that crises harbor are both opportunities for emancipation, democratization, and justice, as well as for exploitation, exclusion, and hostility. This makes “crisis” such an iridescent political concept.
